# Current Concepts of Antigen Cross-Presentation

**DOI:** 10.3389/fimmu.2018.01643

**Published:** 2018-07-16

**Authors:** Maria Embgenbroich, Sven Burgdorf

**Affiliations:** Life and Medical Sciences (LIMES) Institute, University of Bonn, Bonn, Germany

**Keywords:** dendritic cells, cross-presentation, antigen processing, endosomes, antigen dislocation

## Abstract

Dendritic cells have the ability to efficiently present internalized antigens on major histocompatibility complex (MHC) I molecules. This process is termed cross-presentation and is important role in the generation of an immune response against viruses and tumors, after vaccinations or in the induction of immune tolerance. The molecular mechanisms enabling cross-presentation have been topic of intense debate since many years. However, a clear view on these mechanisms remains difficult, partially due to important remaining questions, controversial results and discussions. Here, we give an overview of the current concepts of antigen cross-presentation and focus on a description of the major cross-presentation pathways, the role of retarded antigen degradation for efficient cross-presentation, the dislocation of antigens from endosomal compartment into the cytosol, the reverse transport of proteasome-derived peptides for loading on MHC I and the translocation of the cross-presentation machinery from the ER to endosomes. We try to highlight recent advances, discuss some of the controversial data and point out some of the major open questions in the field.

## Introduction

Dendritic cells (DCs) scan the peripheral tissue for antigens. Upon their recognition, antigens are internalized and the DCs activated and migrate toward the draining lymph node, where they can induce an adaptive immune response (1). In order to do so, they need to process the internalized antigens and load antigen-derived peptides on major histocompatibility complex (MHC) molecules. Peptides loaded onto MHC II molecules can be recognized by antigen-specific CD4^+^ T helper cells. Similarly, peptides loaded on MHC I molecules can be recognized by antigen-specific CD8^+^ T cells, leading to their proliferation and the activation of their cytotoxic capacities.

The presentation of internalized antigens on MHC I molecules is a process termed cross-presentation. Efficient cross-presentation has been shown to be crucial in, e.g., the induction of an adaptive immune response against tumors and viruses that do not infect DCs directly and in the induction of peripheral tolerance (2–5).

The molecular mechanisms that regulate classical antigen presentation on MHC II molecules and cross-presentation, however, have been shown to be quite divers. For MHC II-restricted presentation, internalized antigens are degraded in endo/lysosomal compartments by proteases such as cathepsins. Newly synthesized MHC II molecules, which are stabilized by binding to the invariant chain (Ii), are transported from the ER toward this compartment, where Ii is degraded by lysosomal proteases, resulting in the binding of only a small peptide fragment (CLIP) to MHC II. Subsequently, CLIP is replaced by antigen-derived peptides by the chaperon HLA-DM (6).

In contrast to MHC II-restricted presentation, the molecular mechanisms regulating cross-presentation are less understood and in part discussed controversially. There seems to be a whole variety of pathways leading to antigen cross-presentation and, despite intensive investigations, the molecular mechanisms and individual contribution of each pathway are rather unclear.

In this review, we try to describe some of the recent advances in cross-presentation, focusing on the major cross-presentation pathways and highlighting some of the controversial observations in the field.

## Cross-Presenting DC Subsets

Although many cells are able to present extracellular antigens on MHC I, DCs are considered to be the most prominent and most relevant cross-presenting cells.

In general, DCs are subdivided into conventional DCs (cDCs) and plasmacytoid DCs (pDCs). cDCs are further classified into cDC1 and cDC2 (7). In mice and human, cDC1 are characterized by the expression of the chemokine receptor XCR1 (7–9) and their development relies on the expression of the transcription factors IRF8 and Batf3 (10–12), whereas the development of cDC2 is mainly regulated by IRF4 (7, 13). Additionally, murine cDC1 express either CD8 (in lymphoid tissues) or CD103 (in non-lymphoid tissues), whereas human cDC1 are characterized by the expression of BDCA-3 (CD141) (8, 14–17).

The cDC1 are generally considered to be potent cross-presenting DCs *in vivo*. Accordingly, in murine lymphoid tissue, soluble and cell-associated OVA are cross-presented by resident CD8^+^ DCs (18–20), whereas soluble and cell-associated antigens in lung (21, 22), intestine, and skin (23–25) are cross-presented by migratory CD103^+^ DCs. Further functional properties of cDC1 are the uptake of apoptotic cells *via* Clec9A/DNGR1 (26–29) and the responsiveness to TLR3 stimulation (30).

cDC1 express high levels of MHC I pathway genes (31), show high intra-endosomal reactive oxygen species (ROS) production and low acidification in endosomes (32, 33), all features of efficient cross-presentation (see below). They express the small GTPase Rac2, which enables the assembly of the NAPDH oxidase complex NADPH oxidase 2 (NOX2), resulting in enhanced ROS production and active alkalization of endosomes (33). Additionally, cDC1 show only marginal expression levels of the C-type lectin Siglec-G, a potent inhibitor of NOX2 (34).

However, the cDC1 are not the only cross-presenting DC population. Many other DC subpopulations, including cDC2, have been shown to cross-present as well (35–39). For human DCs, it even has been demonstrated that BDCA3^+^ (cDC1s), BDCA1^+^ (cDC2s), and even pDCs all bear intrinsic capacities to cross-present extracellular antigens (40). The exact role of different cDC1 and cDC2 subpopulations in cross-presentation is, therefore, under debate and, especially since functional data on the physiological role of human DC subsets in cross-presentation is hard to obtain, future experiments will have to shed light on this question.

Although pDCs have been shown to be able to cross-present antigens (41), their role in cross-presentation *in vivo* is questionable, especially since their depletion did not affect cross-presentation and clearance of viral antigens (42).

## Major Pathways of Antigen Cross-Presentation

**Figure 1:**
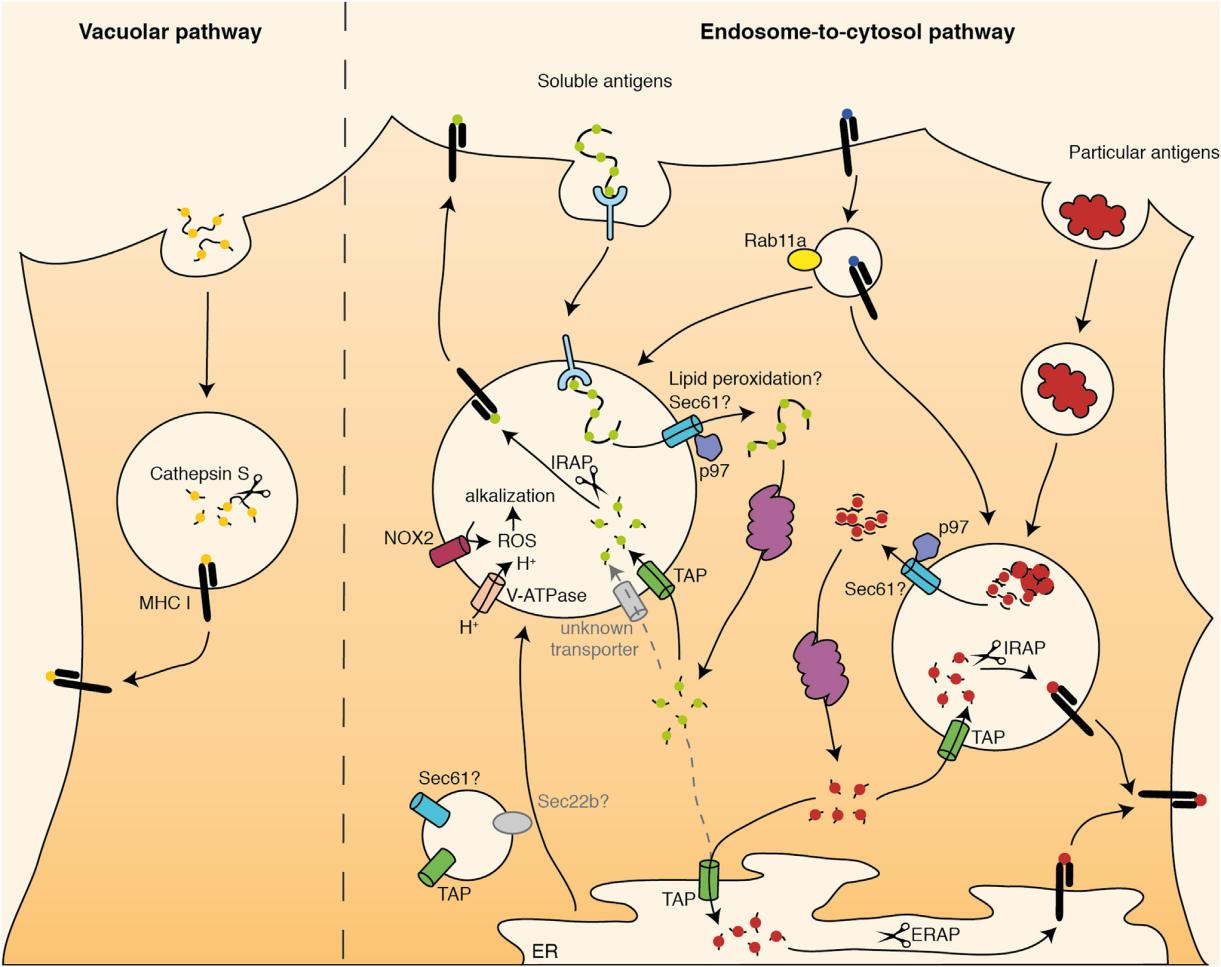
Schematic overview of cross-presentation pathways. Internalized antigens can be presented *via* the vacuolar pathway or *via* the endosome-to-cytosol pathway. In the vacuolar pathway, antigens are degraded in endosomes by Cathepsin S and loaded onto major histocompatibility complex (MHC) I there. In the endosome-to-cytosol pathway, antigens are transported into the cytosol for proteasomal degradation. Afterward, antigen-derived peptides are transported back into the endosomes (soluble and particular antigens) or into the ER (particular antigens) *via* TAP. There, they are trimmed by IRAP (endosomes) or ERAP (ER) and loaded onto MHC I. They cross-presentation machinery might be translocated toward endosomes *via* Sec22b.

Intensive research has clearly shown that there are a wide variety of mechanisms by which peptides derived from extracellular antigens can be presented on MHC I molecules. In general, there are two main cross-presentation pathways: the vacuolar pathway and the endosome-to-cytosol pathway (Figure 1). In the vacuolar pathway, antigen processing and loading onto MHC I molecules occurs within the endo/lysosomal compartment. After internalization, antigens are degraded by lysosomal proteases and antigen-derived peptides are loaded onto MHC class I molecules there. The lysosomal protease Cathepsin S has been demonstrated to play a crucial role in antigen degradation for the vacuolar pathway (43). In the endosome-to-cytosol pathway, internalized antigens need to be transported from the endosomal compartment into the cytosol, where they are degraded by the proteasome (44–46). Derived peptides are subsequently transported by the transporter associated with antigen processing (TAP) into the ER or back into the antigen-containing endosomes, where they can be loaded onto MHC class I (44, 45, 47–49). Although substantial evidence points out that some antigens indeed can be cross-presented independent of proteasomal degradation and TAP-mediated peptide transport by the vacuolar pathway (43, 50–53), most cross-presentation studies report of cross-presentation *via* the endosome-to-cytosol pathway. The dependency of cross-presentation on proteasomal degradation seems logical, since the functional outcome of cross-presentation is the activation of antigen-specific cytotoxic T cells. After migration toward to site of infection, these T cells are fully equipped to kill potential target cells, like virus-infected cells or tumor cells. In order to become functionally active, T cells must recognize the same epitope presented on MHC I by the target cells. Importantly, MHC I-loaded peptides on target cells do not emerge from cross-presentation but rather are the result of direct (classical) MHC I-restricted presentation of endogenous antigens, in which peptides are generated by the proteasome. Since it is hard to assume that for all antigens, the proteasome and lysosomal proteases generate exactly the same epitopes, the dependency of cross-presentation on proteasomal degradation for at least a substantial part of the antigens might circumvent this problem. Accordingly, DCs deficient in the LMP7 subunit of the immunoproteasome are impaired in cross-presentation *in vitro* and *in vivo* (46). However, it needs to be mentioned that very few information about the *in vivo* significance of the vacuolar vs. endosome-to-cytosol pathway is available, pointing out that future experiments are needed to further investigate the relative importance of both pathways *in vivo*.

## Delayed Antigen Degradation and its Role in Cross-Presentation

Over the last years, it has become clear that intra-endosomal antigen stability critically regulates cross-presentation, which efficiency is negatively affected by rapid lysosomal degradation of internalized antigens (54). Lysosomal maturation and activation of lysosomal proteases is fine-tuned by the transcription factor TFEB, an important regulator of cross-presentation (55).

It is generally assumed that rapid antigen degradation quickly destroys a large amount of epitopes before they can be processed properly and loaded onto MHC I molecules (56, 57). Additionally, peptide-loaded MHC I molecules have a limited life span at the cell membrane (58–60). In order to enable T cell activation after migration toward the draining lymph node, however, prolonged cross-presentation seems to be essential. Therefore, limited antigen degradation in antigen-presenting cells might be a mechanism to generate a kind of intracellular antigen depot, from where continuous antigen processing and presentation might ensure the presence of peptide-loaded MHC I molecules over a longer period of time (61). Such intracellular antigen storage depots have also been shown in human monocytes, which accumulate long-peptide antigens for over 5 days in non-lysosomal compartments, where day are protected from rapid degradation (62).

Since DCs are the most efficient cross-presenting cells, these cells possess several mechanisms by which they can actively prevent rapid lysosomal antigen degradation.

First, it was demonstrated that DCs express lower levels of lysosomal proteases (63) and display a reduced velocity of endosome maturation (64) compared to other immune cells. Expression of asparagine endopeptidase and Cathepsins L, S, D, and B in phagosomes of DCs was clearly reduced compared to macrophages, resulting in impaired phagolysosomal degradation and prolonged antigen stability after internalization by DCs (63). The delivery of lysosomal proteases toward phagosomes was even further reduced after stimulation of DCs with LPS (64).

Second, in DCs, an active alkalization of endosomes prevents pH-dependent activation of lysosomal proteases. During lysosome maturation, protons are transported into the luminal space by the V-ATPase, leading to the activation of pH-dependent lysosomal proteases. Reduced V-ATPase activity in DCs might contribute to prevent a rapid drop in pH after antigen internalization (65). Additionally, DCs seem to have the unique capacity to alkalize their endosomes by the recruitment the NOX2 toward the endosomal membrane (32, 66). There, NOX2 can mediate the generation of ROS, which in turn capture protons to build hydrogen peroxide (Figure 1). Proton trapping by ROS causes an active alkalization, impairing pH-dependent activation of lysosomal proteases, which in turn prevents rapid antigen degradation and stimulates cross-presentation (32). The recruitment of NOX2 toward endosomes is mediated by Rab27a (67).

Third, DCs express endocytosis receptors that specifically target non-degradative endosomal compartments. Previous studies have demonstrated that the endocytosis receptor used to internalize an antigen critically determines its intracellular routing and degradation (68). A previous study from our group demonstrated that antigens internalized by fluid phase pinocytosis or scavenger receptor-mediated endocytosis are rapidly targeted toward lysosomes, where they are efficiently degraded by lysosomal proteases, resulting in poor cross-presentation (68). However, if the same DCs simultaneously internalized the same antigen by the mannose receptor, it was targeted toward a distinct pool of early endosomes, which did not undergo rapid fusion with lysosomes and in which antigens were protected from lysosomal degradation, resulting in efficient cross-presentation of MR-internalized antigens (68). Although the role of the MR in *in vivo* cross-presentation has been discussed controversially (69, 70), it now is clear that CD103^+^ DCs in liver and lung use this receptor for cross-presentation of, e.g., viral antigens (71). A correlation between antigen targeting into early endosomes clearly distinct from lysosomes and cross-presentation efficiency has also been confirmed in human DCs. Also in these cells, MR-mediated internalization resulted in its routing into early endosomes, retarded degradation and efficient cross-presentation, whereas uptake by DEC205 lead to antigen targeting into lysosomes, rapid lysosomal degradation, and hence poor cross-presentation (56). Interestingly, attenuating lysosomal degradation was sufficient to rescue the cross-presentation of DEC-205-internalized antigens (56), highlighting again the importance of intra-endosomal antigen stability for efficient cross-presentation. Additionally, the targeted region of the endocytosis receptor might also play a role in antigen degradation and presentation, adding even an additional degree of complexity. Figdor and colleagues demonstrated that antigen targeting toward the carbohydrate recognition domain of DC-SIGN delivers antigens to lysosomal compartments, resulting in rapid degradation and poor cross-presentation, whereas targeting the neck region of DC-SIGN causes antigen delivery in early endosomal compartments clearly distinct from lysosomes, causing prolonged stability and efficient cross-presentation (72, 73).

## Antigen Translocation into the Cytosol as Critical Step in Antigen Cross-Presentation

After being internalized into a non-degradative endosomal compartment, antigens need to be processed before they can be loaded onto MHC I. In the endosome-to-cytosol pathway, internalized antigens, therefore, need to be transported across the endosomal membrane into the cytosol for proteasomal degradation. Although this is a key step in antigen cross-presentation and significant efforts have been made to shed light on this process, the underlying mechanisms mediating such intracellular antigen transport are still topic of debate.

In general, if DCs enable access of endosomal antigens to the cytosol, this must be a process, which is controlled very tightly. Uncontrolled lysosome leakage would lead to the cytosolic release of Cathepsins, which in turn would activate the NLRP3 inflammasome (74) and result in pyroptosis, an inflammatory form of cell death (75). To avoid this, total lysosomal content cannot just be released into the cytosol in an uncontrolled fashion. Accordingly, antigens need to be unfolded (76) and disulfide bridges need to be reduced by the γ-interferon-inducible lysosomal thiol reductase GILT (77) before efficient translocation and hence cross-presentation can take place. This supports the idea that antigen translocation is highly regulated, might involve dislocation through a transmembrane pore complex, and is presumably not the result of simple lysosome leakage.

It is generally assumed that members of the ER-associated degradation (ERAD) machinery contribute in enabling antigen dislocation for cross-presentation. First indirect indications for a role of ERAD in this process came from observations describing the presence of ERAD components in the phagosomal membrane (47, 48) and from experiments using the ERAD inhibitor Exotoxin A, which specifically represses cross-presentation (49, 78).

First direct evidence for the involvement of the ERAD machinery came from the Cresswell group, who demonstrated an important role of the AAA ATPase p97 in antigen dislocation (49). Whereas expression of a dominant-negative p97 mutant in DCs represses cross-presentation, the addition of purified wild-type p97 but not the dominant-negative mutant to purified phagosomes enhanced antigen translocation (49, 79–81), indicating that p97 indeed might provide the energy to pull endosomal antigens into the cytosol.

The identification of a dedicated translocon, which actually functions as a transmembrane pore complex to enable antigen dislocation across the endosomal membrane, has been (and still is) by far more difficult. One putative candidate, which has been proposed to mediate antigen dislocation into the cytosol over a decade ago, is the ERAD member Sec61 (49, 78), a trimeric protein whose downregulation has been shown to inhibit antigen translocation and cross-presentation (79, 82). However, since Sec61 plays an important role in the dislocation of proteins at the ER membrane, like, e.g., the dislocation of MHC I molecules themselves (83), it is very hard to distinguish endosome-specific effects of Sec61 from general effects at the ER. In an attempt to solve this problem, we generated Sec61-specific intracellular antibody (intrabody), which we fused to an ER retention signal (84), leading to the trapping of Sec61 in the ER and preventing its transport toward endosomes (82). By this means, we could demonstrate that the transport of Sec61 toward endosomes indeed is essential for antigen dislocation and cross-presentation. Additionally, we could demonstrate that the expression of the intrabody did not alter overall Sec61 expression and did not affected the ERAD-mediated dislocation of MHC I, TCR, CD3δ and the split venus protein at the ER membrane. This points out that ERAD activity at the ER remained unaltered by the expression of the intrabody. These data suggest that Sec61 indeed might serve as a translocon for cross-presentation (Figure 1). However, it cannot be formally excluded that the translation of another pore complex at the ER membrane is changed by manipulating intracellular Sec61 transport or that another putative pore complex is translocated toward endosomes in a complex with Sec61, being influences by intrabody expression. Additionally, Grotzke et al. demonstrated that a chemical inhibitor of Sec61, mycolactone, does not seem to influence antigen dislocation from the cytosol (85). This inhibitor has been shown to directly bind Sec61 and targets proteins that are co-translationally imported in a Sec61-mediated fashion into the ER toward proteasomal degradation (86, 87). However, whether mycolactone could possibly affect Sec61-mediated protein dislocation from the ER into the cytosol is not clear, especially since such proteins are generally ubiquitinated and targeted for proteasomal degradation also in the absence of mycolactone (80). Indeed, mycolactone was shown to have no influence on ERAD (86) and also Grotzke et al. demonstrated that mycolactone does not affect protein retranslocation from the ER into the cytosol. Whether this is due to a missing role of Sec61 in this process or to specific properties of the inhibitor needs to be determined. Especially since addition of Exotoxin A, an inhibitor that blocks Sec61 channel openings (78, 88), clearly affects antigen translocation and cross-presentation (49, 82), there seems to be a need of information on the exact working mechanism of these inhibitors and on the role of Sec61 in dislocation from the ER to finally clear a potential role of Sec61 on cross-presentation.

In addition to antigen dislocation through a pore complex, a recent study postulated that lipid peroxidation in DCs might play a crucial role in antigen transport into the cytoplasm (89). Here, the authors proposed that the specific recruitment of NOX2 might cause lipid peroxidation in endosomes. As mentioned above, NOX2 captures protons to generate hydrogen peroxide, preventing rapid acidification of the endosome. Lipid peroxidation caused by such hydrogen peroxide was suggested to result in leakiness of the endosomal membrane and hence, antigen access into the cytosol and enhanced cross-presentation. However, it remains unclear how the antigen-presenting cell in this case would prevent inflammasome-induced cell death caused by unspecific release of cathepsins. Additionally, the necessity for endosomal antigens to be unfolded (76) and reduced by GILT (77) cannot be explained by simple leackage of the endosomal membrane. Therefore, the significance of such a pathway in cross-presentation *in vivo* remains to be elucidated.

## Transport of Proteasome-Derived Peptides for Loading Onto MHC I

After being transported into the cytosol, internalized antigens are degraded by the proteasome. Subsequently, antigen-derived peptides can be transported through the TAP transporter into the ER or alternatively, by endosomal TAP, back into the endosomal compartments (44, 45, 47, 48, 90). There, peptides are trimmed into a suited size for loading onto MHC I molecules. Such trimming can occur *via* the peptidases ERAP (in the ER) or IRAP (in endosomes) (Figure 1) (91). Presentation of peptides derived from soluble antigens is mainly ERAP-independent *in vitro* and *in vivo* (92), but rather occurs in endosomes after transport by endosomal TAP and IRAP-mediated peptide trimming (90, 92). Proteasome-derived peptides derived from particulate antigens, however, can be transferred into both the ER and endosomes, where they are trimmed by ERAP or IRAP, respectively, and loaded onto MHC I (91). These underlying mechanisms for these differences are unknown.

Recently, it was demonstrated that, in addition to antigen translocation through endosomal TAP, some peptides might enter endosomes in an energy-consuming but TAP-independent fashion (93), pointing out the possibility of additional (unknown) transporters involved in peptide transport into endosomes for cross-presentation. Additionally, since TAP-independency was often used to demonstrate cross-presentation *via* the vacuolar pathway, there is the possibility that at least in part of these studies, antigens might have entered the endosome *via* the endosome-to-cytosol pathway, using alternative peptide transporters.

After peptide reimport into the endosomes, they can be loaded onto MHC I molecules. In general, there are two basic possibilities how MHC I molecules can enter the endosome. First, newly synthesized MHC I molecules could be transported from the ER to the endosomes and used for peptide loading in cross-presentation. Second, MHC I from the cell surface (that are already loaded with peptides) could be transported toward endosomes during endocytosis events. The Blander group demonstrated that for particulate antigens, MHC I molecules used for cross-presentation mainly originated from the cell membrane and were translocated into an endosomal recycling compartment in a Rab11a-dependent fashion (Figure 1) (94). From these organelles, MHC I molecules can be transported toward phagosomes, a process that is mediated by the SNARE protein SNAP23 and critically depends on MyD88 signaling (94). It remains unclear, however, whether peptide exchange on recycling MHC I molecules requires the help of additional chaperon proteins (similar to the function of HLA-DM in MHC II-restricted presentation) or can occur after simple weakening of the peptide–MHC I binding in endosomes. Additionally, it has been demonstrated that for cross-presentation of elongated peptides, a substantial part of the used MHC I molecules are newly synthesized molecules recruited from the ER (95). In this case, it needs to be determined whether such MHC I molecules are loaded with peptides in the ER and undergo peptide exchange in acidic endosomes, or whether the transport of the entire peptide loading complex, which stabilizes unbound MHC I molecules and assists in peptide binding, to the endosomes is required for cross-presentation. Despite the presence of several members of the peptide loading machinery in endosomes (45, 47, 48), a functional relevance of these proteins in cross-presentation is missing.

## Transport of ER Components to Endosomes

As described above, efficient cross-presentation requires the transport of ER proteins toward endosomes. The exact mechanisms, by which this transfer occurs, are not completely understood and in part contradictory data complicate a clear view on this process.

Since it is known that endosomes during their maturation directly interact with the ER to exchange a wide variety of molecules (96), such ER-endosome membrane contact sites would offer an easy explanation for the transfer of ER proteins toward endosomes. However, it has been proposed by Amigorena and Savina that the transport of cross-presentation components toward endosomes takes place from the ER-golgi intermediate compartment (ERGIC) (97). Membrane fusion between the ERGIC and the phagosomes has been postulated to be mediated by the SNARE proteins Sec22b (in the ERGIC) and syntaxin 4 (in the phagosome). Accordingly, shRNA-mediated downregulation of Sec22b resulted in impaired recruitment of ER proteins toward phagosomes, decreased antigen translocation into the cytosol, and hence reduced cross-presentation (94, 97). These observations support a critical role of the ERAD machinery in antigen dislocation into the cytosol for cross-presentation as described above. However, a recent study by Reddy and colleagues demonstrated that severe off target effects of the used shRNA might have caused the observed influence on cross-presentation (98), questioning the role of Sec22b in cross-presentation. Since such off target effects of shRNA molecules can be circumvented by the generation of Sec22b-deficient mice, one could expect that the use of conditional knockout mice would shed light on the situation and would clearly indicate whether Sec22b is indeed involved in cross-presentation. Mice bearing a conditional knockout of Sec22b in CD11c^+^ DCs were generated by both the Reddy and the Amigorena group. Strikingly, whereas Reddy et al. reported complete independency of cross-presentation on Sec22b (98), Amigorena et al. showed a clear impairment of cross-presentation in Sec22b-knockout DCs, hence drawing completely opposite conclusions (99). Both groups used partially different *in vitro* and *in vivo* systems to substantiate their findings, but since also opposite effects of Sec22b on cross-presentation using the same cells (BM-DCs and splenic DCs) and the same antigens (soluble and bead-bound OVA) were observed, these contractionary results cannot be explained by different experimental setups only (100). Therefore, the exact role of Sec22b and ERGIC-mediated transport of ER proteins needs to be confirmed.

The recruitment of MHC I molecules toward antigen-containing phagosomes was shown to be induced by TLR ligands. TLR-induced and MyD88-dependent signaling resulted in the activation of IKK2, which phosphorylates SNAP23, mediating fusion events between phagosomes and MHC I-containing recycling endosomes (94). Also the transport of other ER proteins toward endosomes has been shown to be stimulated by TLR ligands (82, 90). Using flow cytometric analysis of individual endosomes (101), have demonstrated before that low amounts of Sec61 are present in endosomes also in the absence of TLR ligands, and that a clear recruitment of Sec61 toward antigen-containing endosomes was induced by LPS (82). Since it is very unlikely that Sec61 is also recruited *via* recycling endosomes, distinct mechanisms might come into play for the transport of these molecules.

One of these mechanisms might rely on the uncoordinated 93 homolog B1 (UNC93B1), which is activated by TLR triggering and mediates the transport of TLRs from the ER toward endosomes (102–104). Interestingly, UNC91B1 has been demonstrated to be critically involved in cross-presentation (105). Although a putative role of UNC93B has also been discussed controversially (106), it now becomes clear that an essential role of UNC93B1 in cross-presentation is based on its interaction with the store-operated-Ca^2+^-entry regulator STIM1. UNC93B1 has been shown to be essential for oligomerization of hence activation of STIM1, which in turn alters local Calcium signaling regulating phago/endosome fusion events (107, 108). Ablation of UNC93B1 impairs antigen translocation into the cytosol and cross-presentation (107). Interestingly, antigen dislocation into the cytosol was impaired despite reduced endosomal antigen degradation, which is generally assumed to increase antigen export from the endosomes. Since UNC93B1 upon TLR stimulation mediates TLR transport from the ER toward endosomes, it, therefore, is thinkable that ER members of the cross-presentation machinery are transported from the ER toward endosomes in a similar UNC93B1-dependent fashion.

Additionally, DC activation by TLR ligands can have other effects on the cross-presentation machinery independent of ER to endosome transport, like the prevention of phagosome fusion with lysosomes and concomitant antigen stabilization (57) or increases in antigen internalization (109).

## Alternative Cross-Presentation Pathways

In all cross-presentation pathways described above, cross-presented antigens entered the DC *via* endocytosis. However, there are some reports indicating that also distinct mechanisms can lead to cross-presentation.

One of these mechanisms is the transport of pre-processed antigens (peptides) from a donor cell to a DC. Such transport can occur *via* direct cell–cell contact, mediated by gap junctions (110, 111). After gap junction-mediated transport from one cell to another, antigen-derived peptides can enter the normal MHC I presentation pathway. Interestingly, the donor cell does not need to be an antigen-presenting cell, offering the possibility that DCs can obtain such peptides directly from infected cells. Infection of melanoma cells with *Salmonella* has been demonstrated to increase the expression of Connexin 43, an important gap junction protein, enabling efficient gap junction-mediated peptide transfer from the infected cell to the DC and hence efficient cross-presentation (112). However, given the limited stability of intracellular peptides (113), the physiological significance of such peptide transfer in cross-presentation remains unclear.

Another alternative cross-presentation pathway is termed cross-dressing, which generally implies that the cross-presenting DC becomes an MHC I molecule, which has already been loaded with an antigen-derived peptide, transferred from a donor cell (114, 115). Similar to gap junction-mediated peptide transfer, such donor cell does not necessarily need to be an antigen-presenting cell, suggesting that DCs can derive peptide-loaded MHC I molecules directly from infected cells or even apoptotic cells. The transfer of loaded MHC I molecules is thought to be mediated by cell–cell contact rather than secretory vesicles (114, 116) and overcomes the need of intracellular antigen processing within the DC. Cross-dressing has been shown to occur *in vivo* (114, 116) and cross-dressed DCs have been shown to activate memory T cells after viral infection (116). Remarkably, in this study, the activation of naive T cells did not depend on cross-dressing (116), offering the possibility that different cross-presentation pathways might be responsible for the activation of different T cell populations or for T cell activation under specific conditions. However, the exact physiological relevance of cross-dressing and especially its contribution compared to the other cross-presentation pathways in specific situations, however, remains to be elucidated.

## Conclusion

Despite intensive research over the last decades, several questions regarding the molecular mechanisms of cross-presentation remain unsolved. How are antigens translocated into the cytosol? How are ER components recruited toward endosomes? What is the role of Sec22b and TLR ligands in this process? And probably most important: which of all these proposed mechanisms holds true *in vivo*? Are different cross-presentation pathways used *in vivo* by distinct cell types or antigens (e.g., particulate vs. soluble), or under different physiological conditions? Without any doubt, the elucidation of the molecular mechanisms underlying cross-presentation *in vivo* bears a high intrinsic potential to optimize various vaccination strategies. Therefore, future investigations will be required to shed more light into the exact pathways of cross-presentation and to solve remaining controversies. The publication of clearly contradicting data might suggest the need for common protocols to perform cross-presentation experiments, in particular in regard to cell culture procedures to generate the often used BM-DCs.

## Author Contributions

ME and SB designed the article and wrote the manuscript.

## Conflict of Interest Statement

The authors declare that the research was conducted in the absence of any commercial or financial relationships that could be construed as a potential conflict of interest.
